# TimeDelay-ARACNE: Reverse engineering of gene networks from time-course data by an information theoretic approach

**DOI:** 10.1186/1471-2105-11-154

**Published:** 2010-03-25

**Authors:** Pietro Zoppoli, Sandro Morganella, Michele Ceccarelli

**Affiliations:** 1 Department of Biological and Environmental Studies, University of Sannio, Benevento, I-82100, Italy; 2 Biogem s c a r l, Institute for Genetic Research "Gaetano Salvatore", Ariano Irpino (Avellino), I-83031, Italy

## Abstract

**Background:**

One of main aims of Molecular Biology is the gain of knowledge about how molecular components interact each other and to understand gene function regulations. Using microarray technology, it is possible to extract measurements of thousands of genes into a single analysis step having a picture of the cell gene expression. Several methods have been developed to infer gene networks from steady-state data, much less literature is produced about time-course data, so the development of algorithms to infer gene networks from time-series measurements is a current challenge into bioinformatics research area. In order to detect dependencies between genes at different time delays, we propose an approach to infer gene regulatory networks from time-series measurements starting from a well known algorithm based on information theory.

**Results:**

In this paper we show how the ARACNE (Algorithm for the Reconstruction of Accurate Cellular Networks) algorithm can be used for gene regulatory network inference in the case of time-course expression profiles. The resulting method is called TimeDelay-ARACNE. It just tries to extract dependencies between two genes at different time delays, providing a measure of these dependencies in terms of mutual information. The basic idea of the proposed algorithm is to detect time-delayed dependencies between the expression profiles by assuming as underlying probabilistic model a stationary Markov Random Field. Less informative dependencies are filtered out using an auto calculated threshold, retaining most reliable connections. TimeDelay-ARACNE can infer small local networks of time regulated gene-gene interactions detecting their versus and also discovering cyclic interactions also when only a medium-small number of measurements are available. We test the algorithm both on synthetic networks and on microarray expression profiles. Microarray measurements concern *S. cerevisiae *cell cycle, *E. coli *SOS pathways and a recently developed network for in vivo assessment of reverse engineering algorithms. Our results are compared with ARACNE itself and with the ones of two previously published algorithms: Dynamic Bayesian Networks and systems of ODEs, showing that TimeDelay-ARACNE has good accuracy, recall and *F*-score for the network reconstruction task.

**Conclusions:**

Here we report the adaptation of the ARACNE algorithm to infer gene regulatory networks from time-course data, so that, the resulting network is represented as a directed graph. The proposed algorithm is expected to be useful in reconstruction of small biological directed networks from time course data.

## Background

In order to understand cellular complexity much attention is placed on large dynamic networks of co-regulated genes at the base of phenotype differences. One of the aims in molecular biology is to make sense of high-throughput data like that from microarray of gene expression experiments. Many important biological processes (e.g., cellular differentiation during development, aging, disease aetiology etc.) are very unlikely controlled by a single gene instead by the underlying complex regulatory interactions between thousands of genes within a four-dimension space. In order to identify these interactions, expression data over time can be exploited. An important open question is related to the development of efficient methods to infer the underlying gene regulation networks (GRN) from temporal gene expression profiles. Inferring, or reverse-engineering, gene networks can be defined as the process of identifying gene interactions from experimental data through computational analysis. A GRN can be modelled as a graph *G *= (*V*, *U*, *D*), where *V *is the set of nodes corresponding to genes, *U *is the set of unordered pair (undirected edges) and *D *is the set of ordered pairs *D *(directed edges). A directed edge *d*_*ij *_from *v*_*i *_to *v*_*j *_is present iff there is a causal effect from node *v*_*i *_to node *v*_*j*_. An undirected edge *u*_*ij *_represents the mutual association between nodes *v*_*i *_and *v*_*j*_. Gene expression data from microarrays are typically used for this purpose. There are two broad classes of reverse-engineering algorithms [1]: those based on the physical interaction approach which aim at identifying interactions among transcription factors and their target genes (gene-to-sequence interaction) and those based on the influence interaction approach that try to relate the expression of a gene to the expression of the other genes in the cell (gene-to-gene interaction), rather than relating it to sequence motifs found in the promoters. We will refer to the ensemble of these influence interactions as gene networks. Many algorithms have been proposed in the literature to model gene regulatory networks [2] and solve the network inference problem [3].

### Ordinary Differential Equations

Reverse-engineering algorithms based on ordinary differential equations (ODEs) relate changes in gene transcript concentration to each other and to an external perturbation.

Typical perturbations can be for example the treatment with a chemical compound (i.e. a drug), or the over expression or down regulation of particular genes. A set of ODEs, one for each gene, describes gene regulation as a function of other genes. As ODEs are deterministic, the interactions among genes represent causal interactions, rather than statistical dependencies. The ODE-based approaches yield signed directed graphs and can be applied to both steady-state and time-series expression profiles [3,4].

### Bayesian Networks

A Bayesian network [5] is a graphical model for representing probabilistic relationships among a set of random variables *X*_*i*_, where *i *= 1, ⋯, *n*. These relationships are encoded in the structure of a directed acyclic graph *G*, whose vertexes (or nodes) are the random variables *X*_*i*_. The relationships between the variables are described by a joint probability distribution *P*(*X*_1_, ⋯, *X*_*n*_). The genes, on which the probability is conditioned, are called the parents of gene *i *and represent its regulators, and the joint probability density is expressed as a product of conditional probabilities. Bayesian networks cannot contain cycles (i.e. no feedback loops). This restriction is the principal limitation of the Bayesian network model [6]. Dynamic Bayesian networks overcome this limitation [7]. Dynamic Bayesian networks are an extension of Bayesian networks able to infer interactions from a data set consisting of time-series rather than steady-state data.

### Graphical Gaussian Model

Graphical Gaussian model, also known as covariance selection or concentration graph models, assumes multivariate normal distribution for underlying data. The independence graph is defined by a set of pairwise conditional independence relationships calculated using partial correlations as a measure of independence of any two genes that determine the edge-set of the graph [8]. Partial cross correlation has been also used to deal with time delays [9].

### Gene Relevance Network

Gene relevance networks are based on the covariance graph model. Given a measure of association and defined a threshold value, for all pairs of domain variables (*X, Y*), association *A(X, Y) *is computed. Variables *X *and *Y *are connected by an undirected edge when association *A(X, Y) *exceeds the predefined threshold value. One of the measures of association is the mutual information (MI) [10,11], one of the information theory (IT) main tools. In IT approaches, the expression level of a gene is considered as a random variable. MI is the main tool for measuring if and how two genes influence each other. MI between two variables *X *and *Y *is also defined as the reduction in uncertainty about a variable *X *after observing a second random variable *Y*. Edges in networks derived by information-theoretic approaches represent statistical dependencies among gene expression profiles. As in the case of Bayesian network, the edge does not represent a direct causal interaction between two genes, but only a statistical dependency. It is possible to derive the information-theoretic approach as a method to approximate the joint probability density function of gene expression profiles, as it is performed for Bayesian networks [12-14].

### Time-Course Reverse Engineering

**Figure 1:**
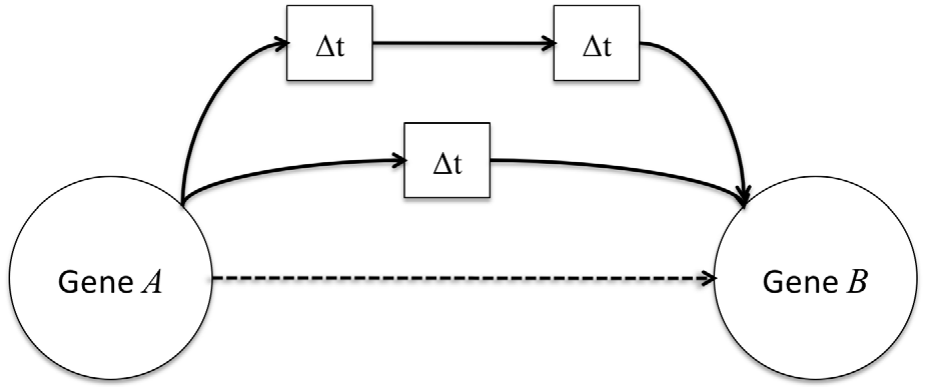
**TimeDelay-ARACNE pairwise time MI idea**. The basic idea of TimeDelay-ARACNE is to represent the time-shifting in the connections rather than unrolling the activation of nodes in time.

Availability of time-series gene expression data can be of help in the study of the dynamical properties of molecular networks, by exploiting the causal gene-gene temporal relationships. In the recent literature several dynamic models, such as Probabilistic Boolean Networks (PBN) [15]; Dynamic Bayesian Networks (DBN) [7]; Hidden Markov Model (HMM) [16] Kalfman filters [17]; Ordinary Differential Equations (ODEs) [4,18]; pattern recognition approaches [19]; signal processing approaches [20], model-free approaches [21] and informational approaches [22] have been proposed for reconstructing regulatory networks from time-course gene expression data. Most of them are essentially model-based trying to uncover the dynamics of the system by estimating a series of parameters, such as auto regressive coefficients [20] or the coefficients of state-transition matrix [17] or of a stiffness matrix [4,18]. The model parameters themselves describe the temporal relationships between nodes of the molecular network. One of the first model-free approaches is reported in [21], where a set classification trees is used in order to learn mutual predictions between time-shifted discrete gene expressions. In particular if a tree is able to predict, at a given accuracy, the activity state of a target gene starting from the activation of another genes, then that tree is considered a regulatory relation. Our work is related to the work of [21] in the sense that it is basically model-free, but it simplifies the method, in the sense that it does not use any prediction model, but evaluates the degree of independence between activations by an information theoretic approach. In addition, several current approaches try to catch the dynamical nature of the network by unrolling in time the states of the network nodes, this is the case of Dynamic Bayesian Networks [7] or Hidden Markov Models [16]. One of the major differences between the approach proposed here and these approaches, is that the dynamical nature of the behavior of the nodes in the networks, in terms of time dependence between reciprocal regulation between them, can be modeled in the connections rather that in the time-unwrapping of the nodes. As reported in Figure 1, we assume that the the activation of a gene *A *can influence the activation of a gene *B *in successive time instants, and that this information is carried out in the connection between gene *A *and gene *B*. Indeed, this idea is also at the basis of the time delay neural network model efficiently used in sequence analysis and speech recognition [23]. Another interesting feature of the reported method, with respect to the ARACNE algorithm, is the fact that the time-delayed dependencies can eventually be used for derive the direction of the connections between the nodes of the network, trying to discriminate between regulator gene and regulated genes. The approach reported here has also some similarities with the method proposed in [22], the main differences are in the use of different time delays, the use of the data processing inequality for pruning the network rather than the minimum description length principle and the discretization of the expression values.

### Summary of the Proposed Algorithm

TimeDelay-ARACNE tries to extend to time-course data ARACNE (Algorithm for the Reconstruction of Accurate Cellular Networks) retrieving time statistical dependency between gene expression profiles. The idea on which TimeDelay-ARACNE is based comes from the consideration that the expression of a gene at a certain time could depend by the expression level of another gene at previous time point or at very few time points before. TimeDelay-ARACNE is a three-steps algorithm: first it detects, for all genes, the time point of the initial changes in the expression, secondly there is network construction and finally a network pruning step. Is is worth noticing that, the analytical tools for time series often require conditions such as stability and stationarity (see [24]). Although it is not possible to state that these conditions hold in general for microarray data, this is due to the limited number of samples and to the particular experimental setup producing the data, nevertheless time series analysis methods have been demonstrated to be useful tools in many applications of time course gene expression data analysis, for example Ramoni *et al*. [25], used an auto-regressive estimation step as feature extraction prior to classification, while Holter *et al*., [26] use the characteristic modes obtained by singular value decomposition to model a linear framework resulting in a time translational matrix. In particular TimeDelay-ARACNE, just as many related works (see for example the paper of [27]) implicitly assumes stationarity and stability conditions in the kernel regression estimator used for the computation of the mutual information, as described in the section Methods. Indeed, the synthetic data generation model (4) and (5) assumes a weakly stationary linear autoregressive time series. We do not attempt removal of the trend because of the short length of the data and the wide variability of the periodicity of the cell division cycle.

## Results and Discussion

### Algorithm Evaluation

TimeDelay-ARACNE was evaluated first alone than against ARACNE, dynamical Bayesian Networks implemented in the Banjo package [28] (a software application and framework for structure learning of static and dynamic Bayesian networks) and ODE implemented in the TSNI package [29] (Time Series Network Identification) with both simulated gene expression data and real gene expression data related to yeast cell cycle [30], SOS signaling pathway in *E. coli *[31] and an in vivo synthetic network, called IRMA [32]. Details on the gene expression data and the construction of the simulated networks are presented in Methods section.

### Synthetic Data

In order to quantitatively evaluate the performance of the algorithm reported here over a dataset with a simple and fair "gold standard" and also to evaluate how the performance depend of the size of the problem at the hand, such as network dimension, number of time points, and other variables we generated different synthetic datasets. Our profile generation algorithm (see Methods) starts by creating a random graph which represents the statistical dependencies between expression profiles, and then the expression values are generated according to a set of stochastic difference equation with random coefficients. The network generation algorithm works in such a way that each node can have zero (a "stimulator" node) one or two regulators. In addition to the random coefficients of the stochastic equations, a random Gaussian noise is added to each expression value. The performance are evaluated for each network size, number of time points and amount of noise by averaging the PPV, recall and *F*-score over a set of 20 runs with different randomly generated networks. The performance is measured in terms of:

• *positive predictive value (PPV)*, it is the percentage of inferred connections which are correct:(1)

• *recall*, it is the percentage of true connection which are correctly inferred by the algorithm:(2)

• *F*-score. Indeed, the overall performance depend both of the positive prediction value and recall as one can improve the former by increasing the detection threshold, but at the expenses of the latter and *vice versa*. The *F*-score measure is the geometric mean of *p *and *r *and represents the compromise between them:(3)

Since TimeDelay-ARACNE always tries to infer edge's direction, so the precision-recall curves take into account direction. As a matter of fact an edge is considered as a true positive only if the edge exist in reference table and the direction is correct.

**Figure 2:**
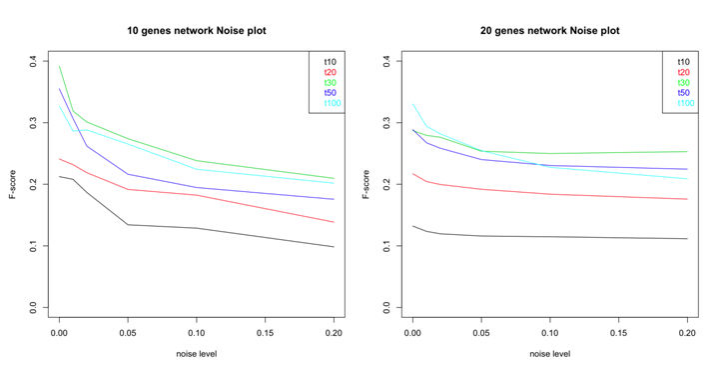
**TimeDelay-ARACNE performance on 10 and 20 genes networks changing noise level**. TimeDelay-ARACNE performance is influenced by noise mostly on 10 genes network. In particular there is a performance decay when the variance of random Gaussian noise increases. In the 20 genes network there isn't the performance decay that we can see in the 10 genes network.

In the first experiment we test TimeDelay-ARACNE performance on different noise levels. We run the algorithm on 2 different network (10 and 20 genes) for 6 different noise levels (random Gaussian noise with zero mean and variance *σ*^2 ^= 0, 0.01, 0.02, 0.05, 0.1, 0.2). As Figure 2 suggest TimeDelay-ARACNE performance is weakly influenced by noise and the performance decay is stronger in the 10 genes network than in the 20 genes network. The *F*-score profile at different noise levels seems to be asymptotic and the performance loss is not more 10%.

We also tested TimeDelay-ARACNE performance on networks with different number of genes and different time points comparing such performances with two other algorithms TSNI, Banjo and the standard ARACNE algorithm. Table 1 and Table 2 show that TimeDelay-ARACNE's performance is only partially directly correlated with time point numbers but inversely with network gene numbers. Probably the information in the end tails of the profiles is not so much easy to detect or perhaps tails became so flat to give an useful information (as often is true with real expression data). TimeDelay-ARACNE performs much better than the two considered algorithms, in addition TSNI probably need a perturbation in order to work better and Banjo needs a very high number of experiments (time points) as compared with the number of genes. As a direct comparison, in these two tables we also report the results in terms of precision and recall of the ARACNE algorithm, although it was not developed for time series we would like to measure the potential improvement of TimeDelay-ARACNE with respect to the standard algorithm. As we can see the standard algorithm has a good precision but a very low recall, this means that even if it is able to correctly recover some true connections, the overall percentage of recovered connections is not enough to obtain a useful *F*-score.

**Table 1:** TimeDelay-ARACNE, TSNI, Banjo and ARACNE performance against synthetic data changing network gene numbers.

		TimeDelay-ARACNE			TSNI			Banjo			ARACNE		
**Genes**	**Time Points**	**PPV**	**Recall**	***F*-score**	**PPV**	**Recall**	***F*-score**	**PPV**	**Recall**	***F*-score**	**PPV**	**Recall**	***F*-score**

genes 10	points 50	0.33	**0.60**	**0.41**	0.43	0.11	0.18	<0.19	0.13	0.15	**0.78**	0.12	0.21

genes 20	points 50	0.46	**0.35**	**0.39**	0.52	<0.1	0.11	<0.1	0.13	0.11	**0.55**	0.06	0.10

genes 30	points 50	0.47	**0.23**	**0.29**	0.35	<0.1	<0.1	<0.1	<0.1	<0.1	**0.68**	0.04	0.08

TimeDelay-ARACNE performance results seem to be correlated with network gene numbers.

**Table 2:** TimeDelay-ARACNE, TSNI, Banjo and ARACNE performance against synthetic data changing network data points.

		TimeDelay-ARACNE			TSNI			Banjo			ARACNE		
**Genes**	**Time Points**	**PPV**	**Recall**	***F*-score**	**PPV**	**Recall**	***F*-score**	**PPV**	**Recall**	***F*-score**	**PPV**	**Recall**	***F*-score**

genes 10	points 10	0.24	**0.22**	**0.22**	**0.29**	0.14	0.19	<0.1	<0.1	<0.1	0.27	0.14	0.17

genes 10	points 20	**0.31**	**0.34**	**0.32**	0.22	0.10	0.14	0.20	<0.1	0.13	0.25	0.09	0.13

genes 10	points 30	0.31	**0.42**	**0.34**	0.39	0.11	0.17	0.17	0.10	0.12	**0.63**	0.14	0.22

genes 10	points 40	0.41	**0.55**	**0.45**	0.39	<0.1	0.15	0.22	0.16	0.18	**0.79**	0.14	0.23

genes 10	points 50	0.33	**0.60**	**0.41**	0.43	0.11	0.18	0.19	0.13	0.15	**0.78**	0.12	0.21

TimeDelay-ARACNE performance results show a partially direct correlation with time points.

### Real Expression Profiles

**Figure 3:**
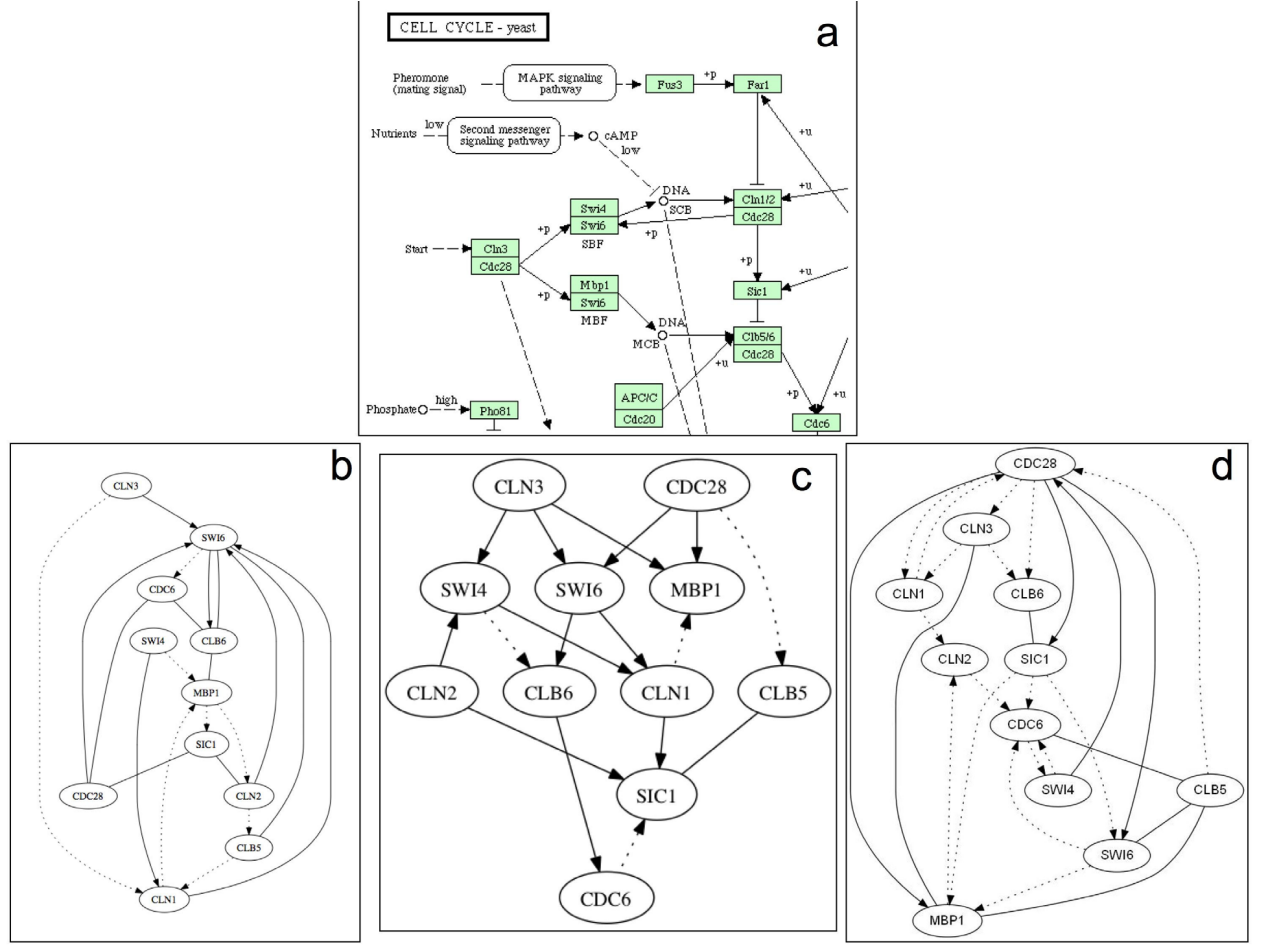
**Yeast cell cycle KEGG pathway and reconstructed network by three different algorithms**. a) is the yeast cell cycle KEGG pathway; b) is the TNSI inferred graph; c) is the TimeDelay-ARACNE inferred graph; d) is the Banjo inferred graph. TSNI and Banjo are used with default settings reported in their manuals. TimeDelay-ARACNE better recover this yeast network topology than other algorithms. Here we represent true positives as straight connections, dotted lines are false positives, false negatives are not reported and considered in the tables of PPV and recall. Missing verse on the connection means that the algorithm recovers the wrong verse.

**Figure 4:**
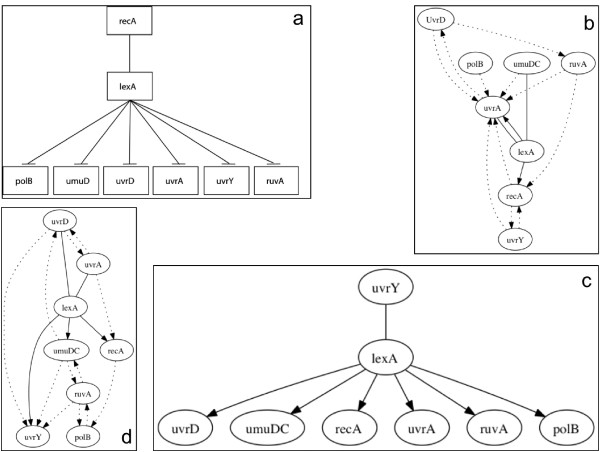
**TimeDelay-ARACNE SOS predicted network and SOS pathway reference**. a) *E. coli *SOS pathway; b) TNSI inferred graph; c) TD-ARACNE inferred graph; d) Banjo inferred graph. TSNI and Banjo are used with default settings reported in their manuals. TD-ARACNE finds *lexA *correctly as the HUB, recovers 6 edges correctly, 1 edge has wrong direction. TD-ARACNE again better recover *E. coli *SOS pathway than other algorithms. Here we represent true positives as straight connections, dotted lines are false positives, false negatives are not reported and considered in the tables of PPV and recall. Missing verse on the connection means that the algorithm recovers the wrong verse.

In order to test TimeDelay-ARACNE performance on expression profiles we selected an eleven genes network from yeast *S. cerevisiae *cell cycle, more precisely part of the G1 step. Selected genes are: *Cln*3, *Cdc*28, *Mbp*1, *Swi*4, *Clb*6, *Cdc*6, *Sic*1, *Swi*6, *Cln*1, *Cln*2, *Clb*5. To try to infer the gene network controlling yeast cell cycle regulation, we choose genes whose mRNA levels respond to the induction of Cln3 and Clb2 that are two well-characterized cell cycle regulators [33]. Late in G1 phase, the Cln3-Cdc28 protein kinase complex activates two transcription factors, MBF (Mbp1 and Swi6) and SBF (Swi4 and Swi6), and these promote the transcription of some genes important for budding and DNA synthesis [34]. Entry into S phase requires the activation of the protein kinase Cdc28p through binding with cyclin Clb5 or Clb6, as well as the destruction of the cyclin-dependent kinase inhibitor Sic1 [35]. Swi4 associates with Swi6 to form the SCB-binding factor complex that activates G1 cyclin genes CLN1 and CLN2 in late G1. Mbp1, a transcription factor related to Swi4, forms the MCB-binding factor complex with Swi6, which activates DNA synthesis genes and S-phase cyclin genes CLB5 and CLB6 in late G1 [36]. In budding yeast, commitment to DNA replication during the normal cell cycle requires degradation of the cyclin-dependent kinase (CDK) inhibitor Sic1. The G1 cyclin-CDK complexes Cln1-Cdk1 and Cln2-Cdk1 initiate the process of Sic1 removal by directly catalyzing Sic1 phosphorylation at multiple sites [37]. In Figure 3 we report network graphs reconstructed by the TimeDelay-ARACNE, TSNI and Banjo. We also report the KEGG pathway of the cell-cycle in yeast. We consider this last information as a true table to compare the results of the algorithm with respect to the others. TSNI and Banjo are used with default settings reported in their manuals. TimeDelay-ARACNE recovers many gene-gene edges as reported in Table 3. We don't use PPV, recall and *F*-score to evaluate the algorithm. Differences between true table and inferred network could be eventually due to the possible incongruence between experimental data and true table. We also tested the proposed algorithm using eight genes by *E. coli *SOS pathway [31]. In the E. coli after the cell is exposed to DNA damaging agents there is the activation of the SOS pathway. Such DNA damaging involves the induction of about 30 genes [38]. Many of these gene products are involved in DNA damage tolerance and repair (e.g. recA, lexA, umuDC, polB, sulA, and uvrA). The SOS response to DNA damage requires the recA and lexA gene products. Near the promoters of the SOS response genes there is a site (the SOS box) bonded by the repressor protein LexA that interferes with the binding of RNA polymerase [39]. Selected genes are: *uvrD*, *lexA*, *umuDC*, *recA*, *uvrA*, *uvrY*, *ruvA*, *polB *as in [40]. In Figure 4 we report the SOS pathway reconstruction by the three algorithms and the relative bibliographic control. In Table 4 there is a detailed description of the eight genes network connections showing that TimeDelay-ARACNE recovers these network topologies better than other algorithms.

**Figure 5:**
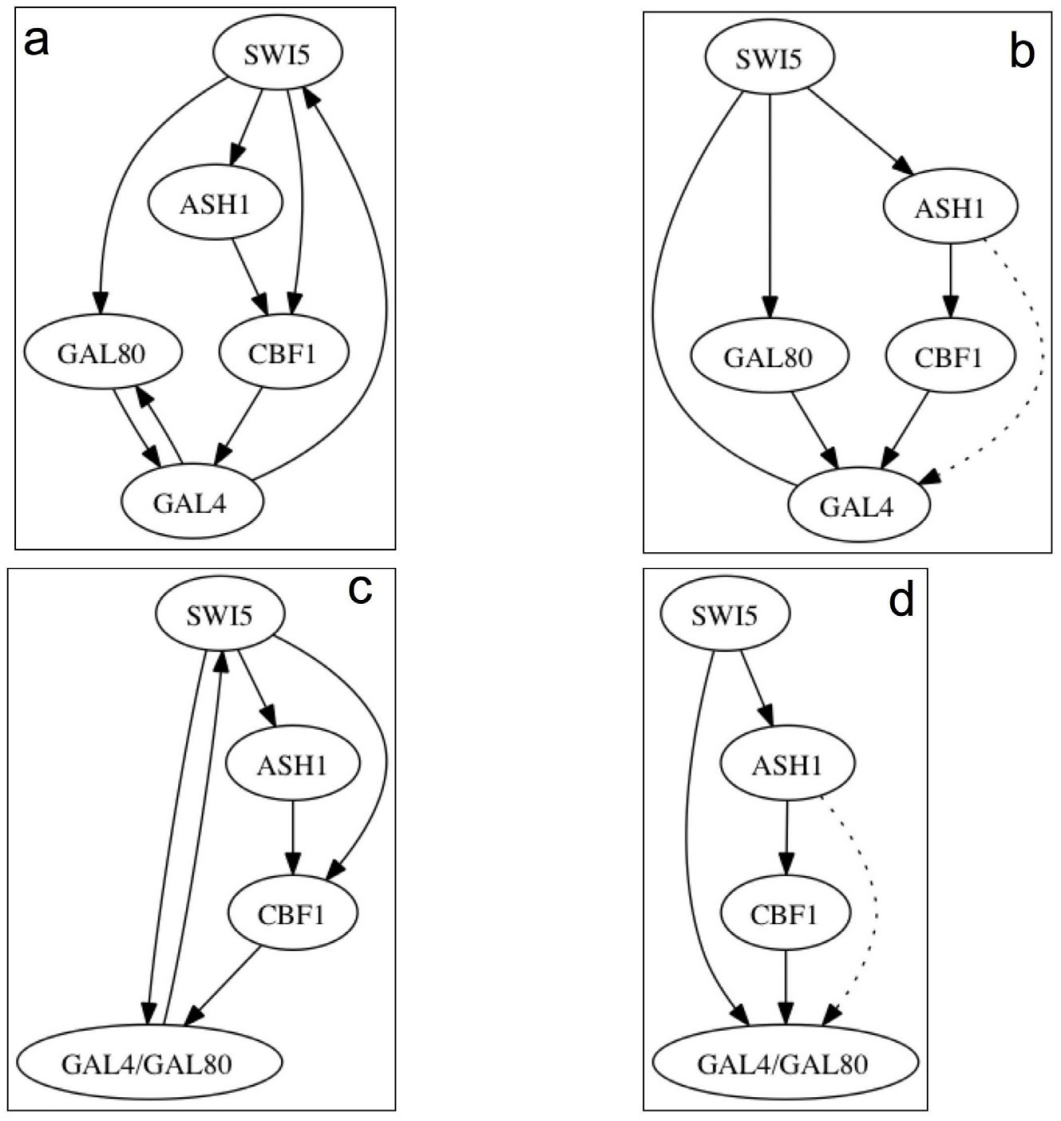
**Yeast Synthetic Network and TimeDelay-ARACNE predicted Yeast Synthetic Network on the Switched ON dataset**. a) the true Yeast Synthetic Network true; b) the TimeDelay-ARACNE inferred graph; c) is the true Yeast Synthetic Network simplified; d) is the TimeDelay-ARACNE inferred graph on simplifed network. In b) TimeDelay-ARACNE correctly recovers 5 edges, 1 edge has wrong direction, 1 is a false positive and 2 edges are missing even if the GAL4→-GAL80 connection is a protein-protein connection like GAL80→GAL4 (which is present anyway) and can't have versus. In d) TimeDelay-ARACNE recovers 4 edges correctly, 1 is a false positive and 2 edges are missing even if GAL4/GAL80→SWI5 connection is a 2 genes auto regulation connection that is not treated in our algorithm. Here we represent true positives as straight connections, dotted lines are false positives, false negatives are not reported. Missing verse on the connection means that the algorithm recovers the wrong verse.

As a further experimental evaluation, we consider a recent significant contribution to system biology given in [32] where the authors built in the yeast *Saccharomyces cerevisiae *a synthetic network, called IRMA, for in vivo "benchmarking" of reverse-engineering and modeling approaches. They tested transcription of network genes upon culturing cells in presence of galactose or glucose. Galactose activates the GAL1-10 promoter, cloned upstream of a Swi5 mutant in the network, and it is able to activate the transcription of all the five network genes. The network is composed of five genes regulating each other; it also is negligibly affected by endogenous genes. The authors measure both time series and steady-state expression data after introducing different perturbations to the network. This is one of the first attempts at building a reference data set having a fair true table. In particular there are two set set of gene profiles called Switch ON and Switch OFF respectively. The former correspond to the shifting of the growing cells from glucose to galactose medium, the latter corresponds to the reverse shift. In Figure 5-a the true IRMA network is reported whereas in Figure 5-b the inferred network by the proposed algorithm is reported. As we can observe, four edges are correctly inferred, one edge has a wrong direction and one is a false positive (Ash1→Gal4) and two edges are missing. (Gal4→Gal80) edge represents a protein-protein connection just like Gal80→Gal4, however this connection in principle cannot have a versus, and this is the reason why the author report a "simplified" network, depicted in figure 5-c, where the complex Gal4/Gal80 is introduced. Again in figure 5-d the inferred network is reported. Here TimeDelay-ARACNE correctly recover four edges, has one false positive and there are two missing edges, however (Gal4/Gal80→Swi5) is a two genes auto-regulation, that is not considered in the developed algorithm. IRMA's data comes out from very strictly controlled experimental conditions so we can use PPV, recall and *F*-score to evaluate the algorithm. The overall results, in terms of PPV, recall and *F*-score, are summarized in table 5. It is to underline that the Switch OFF data are a challenge. There is to take in account the lack of a great stimulus as in the switch ON data. In the Switch OFF condition correlation between genes is obviously less evident, so if we have used the bootstrapped MI as threshold, it would surely overcome any signal.

**Table 3:** TimeDelay-ARACNE test on the yeast eleven genes network.

Genes	Kegg	Correct	Wrong
*Cln3*	*Swi4, Swi6, Mbp1*	*Swi4, Swi6, Mbp1*	-

*Swi6*	*Cln1/2, Clb5/6*	*Cln1, Clb6*	*Clb5*

*Swi4*	*Cln1/2, CDC28*	*Cln1*	*Clb6*

*Mbp1*	*Clb5/6, Cdc28*	*-*	-

*Cln1*	*Sic1*	*Sic1*	-

*Cln2*	*Sic1, Swi4/6*	*Sic1, Swi4*	-

*Clb5*	*Cdc6*	*-*	*Sic1*

*Clb6*	*Cdc6*	*Cdc6*	-

*Sic1*	*Clb5/6, Cdc28*	*-*	-

*Cdc6*	*-*	*-*	*Sic1*

*Cdc28*	*Swi4/6, Mbp1, Cln1/2, Sic1, Cdc6*	*Swi6, Mbp1*	*Clb5*

It is important to underline that *Cdc28 *is the only yeast Cyclin-dependent kinase and it is present during the whole cell cycle. From the KEGG pathway it is clear that *Cdc28 *makes complex with cyclins (*Cln *and *Clb*) but these complexes are of course at proteomic level and they aren't related to the *Cdc28 *transcription but mostly to the cyclins transcript levels. According to this, TimeDelay-ARACNE misconnections couldn't be considered errors.

**Table 4:** TimeDelay-ARACNE test on the *E. coli *eight genes network.

Genes	SOS True Relations	Correct	Wrong
*recA*	*lexA*	-	-

*lexA*	*uvrD, umuDC, recA, uvrA, uvrY, ruvA, polB.*	*uvrD, umuDC, recA, uvrA, ruvA, polB.*	-

*uvrY*	-	-	*lexA*

TimeDelay-ARACNE correctly infers the *lexA *to *recA *edge but can not infer *recA *to *lexA *edge due a limitation of the algorithm. TimeDelay-ARACNE also infers *lexA *to *uvrY *edge but the direction is wrong.

**Table 5:** TimeDelay-ARACNE test on the IRMA in vivo synthetic network.

	TimeDelay-ARACNE			TSNI			Banjo			ARACNE		
	**PPV**	**Recall**	***F*-score**	**PPV**	**Recall**	***F*-score**	**PPV**	**Recall**	***F*-score**	**PPV**	**Recall**	***F*-score**

	Switch ON network											

**5 genes network**	0.71	**0.67**	**0.69**	**0.80**	0.50	0.61	0.30	0.25	0.27	0.5	0.6	0.54

**simplified network**	0.80	**0.67**	0.73	**1.0**	**0.67**	**0.80**	-	-	-	0.5	0.5	0.5

	Switch OFF network											

**5 genes network**	0.37	**0.60**	**0.46**	**0.60**	0.38	**0.46**	0.6	0.38	0.46	0.25	0.33	0.28

**simplified network**	0.50	**0.75**	**0.60**	**0.75**	0.5	**0.60**	-	-	-	0.5	0.6	0.54

Accuracy of the considered algorithm on the dataset of the IRMA network.

According to this we try a compromise: no threshold was applied but we just apply the DPI pruning. We can observe that TimeDelay-ARACNE reaches good performance, in terms of recall and *F*-score, in all considered cases with respect to the results reported in [32] and to the ARACNE results over the same networks. This means that it reaches a good compromise between PPV and recall.

## Conclusions

The goal of TimeDelay-ARACNE is to recover gene time dependencies from time-course data producing oriented graph. To do this we introduce time Mutual Information and Influence concepts. First tests on synthetic networks and on yeast cell cycle, SOS pathway data and IRMA give good results but many other tests should be made. Particular attention is to be made to the data normalization step because the lack of a rule. According to the little performance loss linked to the increasing gene numbers shown in this paper, next developmental step will be the extension from little-medium networks to medium networks.

## Methods

### Datasets

#### Simulated Gene Expression Data

We construct some synthetic gene networks in order to compute the functions *p*, *r *and *F*-score of the method having reference true tables and to compare its performance to other methods. According to the terminology in [41] we consider a gene network to be well-defined if its interactions allow to distinguish between *regulator *genes and *regulated *genes, where the first affect the behaviour of the second ones. Given a well defined network, we can have genes with zero regulators (called *stimulators*, which could represent the external environmental conditions), genes with one regulator, genes with two regulators, and so on. If a gene has at least one regulator (it is not a stimulator) then it owns a regulation function which describes its response to a particular stimulus of its regulator/regulators.

Our synthetic networks contain some stimulator genes with a random behaviour and regulated genes which can eventually be regulators of other genes. The network dynamics are modeled by linear causal relations which are formulated by a set of randomly generated equations. In particular, let us call the expression of gene *i *at time *t *as , our synthetic network generation module works as follows,

• if gene *i *is a stimulator gene then its expression profile, , *t *= 0, 1, ... is randomly initialized with a sequence of uniform numbers in [1, 100].

• for each non-stimulator gene *i*,  is initialized with a uniform random number in [1,100]

• for each non-stimulator gene *i*, the expression values , ...,  are computed according to a stochastic difference equation with random coefficients depending on one or two other regulator genes by using one of the two equations below:(4)

here the coefficients  and  are random variables uniformly distributed in [0, 1] and  is a random Gaussian noise with zero mean and variance *σ*^2^. Moreover the regulators genes *p*_*i *_and *q*_*i *_of the *i*-th are randomly selected at the beginning of each simulation run. The network generation algorithm is set in such a way that 75% of genes have one regulator and 25% of genes have two regulators.

• each expression profile is then normalized to be within the interval [0, 1]

In our experiments, the PPV, recall and *F*-score of the proposed and the other methods is computed as the average over a set of 20 runs over different random networks with the same number of genes, number of time points and noise levels.

#### Microarray Expression Profiles

The time course profiles for a set of 11 genes, part of the G1 step of yeast cell cycle, are selected from the widely used yeast, *Saccharomyces cerevisiae*, cell cycle microarray data [30]. These microarray experiments were designed to create a comprehensive list of yeast genes whose transcription levels were expressed periodically within the cell cycle. We select one of this profile in which the gene expressions of cell cycle synchronized yeast cultures were collected over 17 time points taken in 10-minute intervals. This time series covers more than two complete cycles of cell division. The first time point, related to the M step, is excluded in order to better recover the time relationships present in the G1 step. The true edges of the underlying network were provided by KEGG yeast's cell cycle reference pathway [42].

#### Green Fluorescent Protein Real-Time Gene Expression Data

The time course profiles for a set of 8 genes, part of the SOS pathway of *E. coli *[31] are selected. Data are produced by a system for real-time monitoring of the transcriptional activity of operons by means of low-copy reporter plasmids in which a promoter controls GFP (green fluorescent protein). Even if the data contain 50 time points we use only the first 14 points (excluding the first point of the TS data which is zero) avoiding the misguiding flat tails characterizing such gene profiles (the response to the UV stimulus is quick, so very soon mRNAs came back to pre-stimulus condition). The expression levels are obtained by averaging the replicates.

#### IRMA network

Two sets of five genes of time course profiles are provided by real-time PCR from an in vivo yeast synthetic network [32]. One set, called Switch ON data set, is the result of the time measurements, every 20 minutes for 5 hours, of the mRNA concentration after shifting cells from glucose to galactose, for a total of 5 profiles of 16 points. The other one, called Switch OFF data set, is the result of the time measurements, every 10 minutes for 3 hours, of the mRNA concentration after shifting cells from galactose to glucose, for a total of 5 profiles of 21 points. The true edges of the underlying network are provided by the experiment design, and are provided as supplementary information from the paper [32].

## Algorithms

### ARACNE

The ARACNE algorithm has been proposed in [12,43]. ARACNE is an information-theoretic algorithm for the reverse engineering of transcriptional networks from steady-state microarray data. ARACNE, just as many other approaches, is based on the assumptions that the expression level of a given gene can be considered as a random variable, and the mutual relationships between them can be derived by statistical dependencies. It defines an edge as an irreducible statistical dependency between gene expression profiles that cannot be explained as an artifact of other statistical dependencies in the network. It is a two steps algorithm: network construction and network pruning. Within the assumption of a two-way network, all statistical dependencies can be inferred from pairwise marginals, and no higher order analysis is needed. ARACNE identifies candidate interactions by estimating pairwise gene expression profile mutual information, *I*(*g*_*i*_, *g*_*j*_) ≡ *I*_*ij*_, an information-theoretic measure of relatedness that is zero iff the joint distribution between the expression level of gene *i *and gene *j *satisfies *P(g*_*i*_, *g*_*j*_*) *= *P*(*g*_*i*_)*P*(*g*_*j*_). ARACNE estimates MI using a computationally efficient Gaussian Kernel estimator. Since MI is reparameterization invariant, ARACNE copula-transforms (i.e., rank-order) the profiles before MI estimation; the range of these transformed variables is thus between 0 and 1, and their marginal probability distributions are manifestly uniform. This decreases the influence of arbitrary transformations involved in microarray data pre-processing and removes the need to consider position-dependent kernel widths which might be preferable for non-uniformly distributed data. Secondly the MIs are filtered using an appropriate threshold, *I*_0 _thus removing the most of indirect candidate interactions using a well known information theoretic property, the data processing inequality (DPI). ARACNE eliminate all edges for which the null hypothesis of mutually independent genes cannot be ruled out. To this extent, ARACNE randomly shuffles the expression of genes across the various microarray profiles and evaluate the MI for such manifestly independent genes. The DPI states that if genes *g*_1 _and *g*_3 _interact only through a third gene, *g*_2_, (i.e., if the interaction network is *g*_1 _↔ ... ↔ *g*_2 _↔ ... ↔ *g*_3 _and no alternative path exists between *g*_1 _and *g*_3_), then *I*(*g*_1, _*g*_3_) ≤ min(*I*(*g*_1_, *g*_2_); *I*(*g*_2_, *g*_3_)) [44]. Thus the least of the three MIs can come from indirect interactions only, and so it's pruned.

### TimeDelay-ARACNE

TimeDelay-ARACNE tries to extend to time-course data ARACNE retrieving time statistical dependency between gene expression profiles. TimeDelay-ARACNE is a 3 steps algorithm: it first detects, for all genes, the time point of the initial changes in the expression, secondly there is network construction than network pruning.

#### Step 1

The first step of the algorithm is aimed at the selection of the initial change expression points in order to flag the possible regulator genes [7]. In particular, let us consider the sequence of expression of gene *g*_*a*_:  we use two thresholds *τ*_*up *_and *τ*_*down *_and the initial change of expression (*IcE*) is defined as(6)

The thresholds are chosen with . In all the reported experiments we used *τ*_*up *_= 1.2 and consequently *τ*_*down *_= 0.83. The quantity *IcE(g*_*a*_*) *can be used in order to reduce the unuseful influence relations between genes. Indeed, a gene *g*_*a *_can eventually influence gene *g*_*b *_only if *IcE(g*_*a*_*) *≤ *IcE*(*g*_*b*_) [7].

#### Step 2

The basic idea of the proposed algorithm is to detect time-delayed dependencies between the expression profiles by assuming as underlying probabilistic model a stationary Markov Random Field [45]. In particular the model should try to catch statistical dependencies between the activation of a given gene *g*_*a *_at time *t *and another *g*_*b *_at time *t *+ *κ *with *Ice*(*g*_*a*_) ≤ *Ice*(*g*_*b*_). Our assumption relies on the fact the probabilistic properties of the network are determined by the joint distribution . Here  is the expression series of gene *g*_*b *_shifted *κ *instants forward in time. For our problem we should therefore try to estimate both the stationary joint distribution  and, for each pair of genes, the best suited delay parameter *κ*. In order to solve these problems, as in the case of the ARACNE algorithm [43], the copula-based estimation can help in simplifying the computations [46]. The idea of the copula transform is based on the assumption that a simple transformation can be made of each variable in such a way that each transformed marginal variable has a uniform distribution. In practice, the transformations might be used as an initial step for each margin [47]. For stationary Markov models, Chen *et al*. [46] suggest to use a standard kernel estimator for the evaluation of the marginal distributions after the copula transform. Here we use the simplest rank based empirical copula [47] as other kind of transformations did not produce any particular advantage for the considered problem. Starting from a kernel density estimation  of *P *the algorithm identifies candidate interactions by pairwise time-delayed gene expression profile mutual information defined as:(7)

Therefore, time-dependent MIs are calculated for each expression profile obtained by shifting genes by one time-step till the defined maximum time delay is reached (see Figure 1, by assuming a stationary shift invariant distribution. After this we introduce the Influence as the max time-dependent MIs, *I*^*κ *^(*g*_*A*_, *g*_*B*_), over all possible delays *κ*:(8)

TimeDelay-ARACNE infers directed edges because shifted gene values are posterior to locked gene ones; so, if there is an edge it goes from locked data gene to the other one. Shifting profiles also makes the influence measure asymmetric:(9)

In particular, if the measure *Infl*(*g*_*a*_, *g*_*b*_) is above the the significance threshold, explained below, for a value of *κ *> 0, then this means that the activation of gene *g*_*a *_influences the activation of gene *g*_*b *_at a later time.

In other terms there is a directed link "from" gene *g*_*a *_"to" gene *g*_*b*_, this is the way TimeDelay-ARACNE can recover directed edges. On the contrary, the ARACNE algorithm does not produce directed edges as it corresponds to the case *κ *= 0, and the Mutual Information is of course symmetric.

We want to show direct gene interactions so under the condition of the perfect choice of experimental time points the best time delay is one because it allows to better capture direct interactions while other delays ideally should evidence more indirect interactions but usually time points are not sharply calibrated to detect such information, so considering few different time points could help in the task. If you consider a too long time delay you can see a correlation between gene *a *and gene *c *losing gene *b *which is regulated by *a *and regulates *c *while short time delay can be not sufficient to evidence the connection between gene *a *and gene *b*, so using some few delays we try to overcome the above problem. Other approaches based, for example, of conditional mutual information, such as in [48], could of course be exploited.

**Figure 6:**
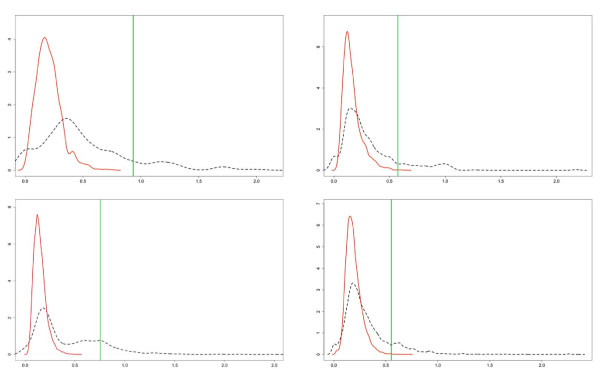
**Block Bootstraping for MI**. We report the distribution (black line) of the bootstrapped MI for networks of 10,20,30 and 50 genes (top to down and from left to right). The figure presents (in green) the thresholds, we also plot (in red) the distribution of the bootstrapped MI of two randomly selected non interacting genes. In general the percentage of values of MI above the threshold is always below the 5%.

After the computation of the *Infl*() estimations, TimeDelay-ARACNE filters them using an appropriate threshold, *I*_0_, in order to retrieve only statistical significant edges. TimeDelay-ARACNE auto-sets this threshold using a stationary bootstrap on the time data. The bootstrap is a method for estimating the distribution of a given estimator or test statistic by resampling available data. The methods that are available for implementing the bootstrap, and the improvements in accuracy that it achieves in terms of asymptotic approximations, depend on whether the data are a random sample from a distribution or a time series [49]. If the data are a random sample (i.i.d. data), then the bootstrap can be implemented by sampling the data randomly with replacement or by sampling a parametric model of the distribution of the data. In [50-53] detailed discussions of bootstrap methods and their properties for data that are sampled randomly from a distribution can be found. The situation is more complicated when the data are a time series because bootstrap sampling must be carried out in a way that suitably captures the dependence structure of the data generation process. The block bootstrap is the best-known method for implementing the bootstrap with time-series data [54]. It consists of dividing the data into blocks of observations and then sampling the blocks at random with replacement. The blocks may be non-overlapping [51,55] or overlapping [56,57] The bootstrap sample is obtained by sampling blocks randomly with replacement and laying them end-to-end in the order samples. It is also possible to use overlapping blocks with lengths that are sampled randomly from the geometric distribution. The block bootstrap with random block lengths is also called the stationary bootstrap because the resulting bootstrap data series is stationary, whereas it is not true with overlapping or non-overlapping blocks of non-stochastic lengths. According to the previous explanation, in order to compute a useful significance threshold of the Mutual Information we implement a stationary bootstrap. First we sample the block length from a random-generated geometric distribution, than we randomly choose the initial position of the block in the time series data. Blocks selected in this way, having different lengths and overlapping, are then concatenated to obtain for each gene a new random time series. Using these new time series, TimeDelay-ARACNE algorithm calculates the "bootstrapped data" MIs. Such procedure is repeated many times in order to have the mean MI, *μ*, and the standard deviation *σ*. The threshold is then set with *I*_0 _= *μ *+ *α*·*σ*. In figure 6 we report the distribution (black line) obtained by boostrapping MI for networks of 10,20,30 and 50 genes. The figure also presents (in green) the thresholds, we also plot (in red) the distribution of the bootstrapped MI of two randomly selected non interacting genes. In general the percentage of values of MI above the threshold is always below the 5%.

#### Step 3

In the last step TimeDelay-ARACNE uses the DPI twice. In particular the first DPI is useful to split three-nodes cliques (triangles) at a single time delay. Whereas the second is applied after the computation of the time delay between each pair of genes as in (8). Just as in the standard ARACNE algorithm, three genes loops are still possible on the basis of a tolerance parameter. In particular triangles are maintained by the algorithm if the difference between the mutual information of the three connections is below the 15% (this the same tolerance parameter adopted in the ARACNE algorithm).

#### Computational Issues

The computational performance of the TimeDelay-ARACNE algorithm is influenced by the number of genes, by the mutual information estimation algorithm and by the adopted scheme of bootstrapping for the estimation of the threshold parameter. In particular if the network has *n *genes and *t *samples, we have to compute *O(Kn*^2^*) *estimations of the mutual information between two vectors of samples having *t *elements or less, *K *being the maximum value of the parameter *κ*. We adopt a kernel-based estimator of the density of data used in the computation of Mutual Information; it is based on procedure proposed in [58] and implemented in the R package "GenKern" http://cran.r-project.org/. It performs a smoothing of data and an interpolation on a grid of fixed dimensions; the procedure also performs an automatic selection of the kernel bandwidth, by choosing the bandwidth which (approximately) minimizes good quality estimates of the mean integrated squared error [59]. Indeed, there are also other, more recent and elaborate, approaches for estimating entropy and Mutual Information. In particular approaches such as those proposes in [11,60] deal with entropy estimation in the cases of a small number of high-dimensional samples, where the kernel-based density estimator could be rather inefficient.

Therefore, each inner mutual information estimation just depends on *t *and on the size of the fixed grid, which in all our experiments we fixed at 100 × 100. The algorithms were developed in R and available at the site http://bioinformatics.biogem.it. To have an idea of the computational time required by each network reconstruction, the estimation of the mutual information on a standard platform (Intel Core 2 **2, 4 GHz **Duo processor with **2 GB RAM**) between two expression profiles of size from 10 to 100 points ranges on the average between 0.07 and 0.13 seconds. The whole procedure, apart from the bootstrapping required to estimate the threshold *I*_0_, on a network of 50 genes and 50 time points, requires less than 7 minutes. Therefore the most computational demanding step is the bootstrapping, it is needed to compute the threshold *I*_0_. It consists in randomly permuting the dataset (the set of expression profiles row values), and then calculating the average mutual information and standard deviation of these random values. Depending on the number of samples in the bootstrap steps, the computational time changes; in all the reported experiments we used a number of 500 bootstrapping samples, this turns out to produce the reconstructed network of 50 genes and 50 time points in about 47 minutes.

## Availability and Requirements

The software was implemented in R and can be downloaded at http://bioinformatics.biogem.it or by contacting the corresponding author.

## Authors' contributions

PZ designed the procedure and discussed the results, SM contributed to the implementation of the procedure and performed the elaboration on data, MC designed the procedure, proposed the biological problem and discussed the results. All authors contributed to the design of the whole work and to the writing of the manuscript. All authors read and approved the final manuscript.
